# Efficacy of polygenic risk scores and digital technologies for INNOvative personalized cardiovascular disease PREVention in high-risk adults: protocol of a randomized controlled trial

**DOI:** 10.3389/fpubh.2024.1335894

**Published:** 2024-06-14

**Authors:** Roberta Pastorino, Angelo Maria Pezzullo, Antonella Agodi, Chiara de Waure, Walter Mazzucco, Luigi Russo, Martina Bianchi, Alessandra Maio, Sara Farina, Martina Porcelli, Diego Maria Tona, Matteo Di Pumpo, Rosarita Amore, Malgorzata Wachocka, Tina Pasciuto, Martina Barchitta, Roberta Magnano San Lio, Giuliana Favara, Antonino Tuttolomondo, Fabio Tramuto, Gaia Morello, Daniele Domenico De Bella, Santo Fruscione, Anna Severino, Giovanna Liuzzo, Stefania Boccia

**Affiliations:** ^1^Section of Hygiene, Department of Life Sciences and Public Health, Università Cattolica del Sacro Cuore, Rome, Italy; ^2^Department of Woman and Child Health and Public Health, Fondazione Policlinico Universitario A. Gemelli IRCCS, Rome, Italy; ^3^Department of Medical and Surgical Sciences and Advanced Technologies “GF Ingrassia”, University of Catania, Catania, Italy; ^4^Azienda Ospedaliero Universitaria Policlinico “G. Rodolico-San Marco”, Programme: Hospital Epidemiology and Translational Research: Epidemiological Surveillance, Risk Assessment and Control, Catania, Italy; ^5^Department of Medicine and Surgery, University of Perugia, Perugia, Italy; ^6^Azienda Ospedaliera Universitaria Policlinico “Paolo Giaccone” di Palermo, Palermo, Italy; ^7^Dipartimento PROMISE, Università degli Studi di Palermo, Palermo, Italy; ^8^Research Core Facilty Data Collection G-STeP, Fondazione Policlinico Universitario A. Gemelli IRCCS, Rome, Italy; ^9^Department of Cardiovascular Sciences, Fondazione Policlinico Universitario A. Gemelli—IRCCS, Rome, Italy; ^10^Department of Cardiovascular and Pulmonary Sciences, Università Cattolica del Sacro Cuore, Rome, Italy

**Keywords:** cardiovascular disease, polygenic risk score (PRS), smart bands, personalized prevention, trial

## Abstract

**Background:**

Cardiovascular diseases (CVDs) pose a significant global health challenge, necessitating innovative approaches for primary prevention. Personalized prevention, based on genetic risk scores (PRS) and digital technologies, holds promise in revolutionizing CVD preventive strategies. However, the clinical efficacy of these interventions requires further investigation. This study presents the protocol of the INNOPREV randomized controlled trial, aiming to evaluate the clinical efficacy of PRS and digital technologies in personalized cardiovascular disease prevention.

**Methods:**

The INNOPREV trial is a four-arm RCT conducted in Italy. A total of 1,020 participants, aged 40–69 with high 10-year CVD risk based on SCORE 2 charts, will be randomly assigned to traditional CVD risk assessment, genetic testing (CVD PRS), digital intervention (app and smart band), or a combination of genetic testing and digital intervention. The primary objective is to evaluate the efficacy of providing CVD PRS information, measured at baseline, either alone or in combination with the use of an app and a smart band, on two endpoints: changes in lifestyle patterns, and modification in CVD risk profiles. Participants will undergo a comprehensive assessment and cardiovascular evaluation at baseline, with follow-up visits at one, five, and 12 months. Lifestyle changes and CVD risk profiles will be assessed at different time points beyond the initial assessment, using the Life's Essential 8 and SCORE 2, respectively. Blood samples will be collected at baseline and at study completion to evaluate changes in lipid profiles. The analysis will employ adjusted mixed-effect models for repeated measures to assess significant differences in the data collected over time. Additionally, potential moderators and mediators will be examined to understand the underlying mechanisms of behavior change.

**Discussion:**

As the largest trial in this context, the INNOPREV trial will contribute to the advancement of personalized cardiovascular disease prevention, with the potential to positively impact public health and reduce the burden of CVDs on healthcare systems. By systematically examining the clinical efficacy of PRS and digital interventions, this trial aims to provide valuable evidence to guide future preventive strategies and enhance population health outcomes.

## Background

Cardiovascular diseases (CVDs) continue to pose a significant global health challenge, representing the leading cause of morbidity and mortality worldwide (1). With their multifactorial nature, CVDs encompass a range of conditions such as coronary artery disease, stroke, and heart failure, affecting millions of individuals and placing an immense burden on healthcare systems (2, 3).

The burden of CVDs is driven by various risk factors, including unhealthy lifestyles, genetic predisposition, and socioeconomic factors. Despite advancements in medical interventions and improved awareness of preventive measures, the incidence and prevalence of CVDs remain substantial, underscoring the need for innovative approaches in primary prevention (1–3).

Personalized prevention, tailored to an individual's unique genetic makeup and lifestyle characteristics, holds great promise in revolutionizing preventive strategies for CVDs. Among the emerging tools in this field, polygenic risk scores (PRS) have gained attention as a potential means of assessing an individual's genetic susceptibility to CVDs. By aggregating information from multiple genetic variants, PRS offers a comprehensive assessment of an individual's genetic risk profile, aiding in the identification of high-risk individuals who may benefit from targeted preventive interventions (4–6).

Additionally, the rapid advancements in digital technologies have opened up new opportunities for personalized healthcare delivery and behavior modification. Ranging from wearable devices monitoring physical activity, dietary, and sleep habits to smartphone applications promoting healthy habits, digital tools provide innovative ways to engage individuals and support sustainable lifestyle changes (7–9).

Despite the increasing interest in utilizing PRS and digital technologies for personalized cardiovascular disease prevention, their clinical efficacy and real-world impact are still to be fully elucidated. Furthermore, the efficacy of communicating the PRS to the person relies on the assumption that receiving genetic feedback will motivate individuals to change their behaviors and reduce their risk of developing the disease. A comprehensive evaluation of these interventions through large-scale prospective studies is necessary to determine their utility, feasibility, and potential benefits in reducing CVD risk at the population level.

Therefore, this paper presents the rationale and design of a randomized controlled trial (RCT) that aims to evaluate the clinical efficacy of PRS and digital technologies in personalized CVD prevention. By examining the impact of these interventions on modifying lifestyle patterns and improving cardiovascular risk profiles, this trial intends to contribute valuable evidence to guide future preventive strategies and enhance population health outcomes.

## Objectives

We will evaluate whether the use of innovative technologies to gather information on lifestyle and genetic determinants in the primary prevention setting can lead to changes in individuals' behaviors and CVD risk. The primary objective is to assess the efficacy of providing CVD polygenic risk score (CVD PRS) information, measured at baseline, either alone or in combination with the use of an app and a smart band (digital technology), on two endpoints: (i) changes in lifestyle patterns, and (ii) modification in CVD risk profiles. The potential lifestyle modification will also be evaluated in relation to other factors such as psychological status, knowledge and attitudes toward CVDs and personalized medicine, and acceptance of wearable devices, which can influence citizens' behavior.

### Hypothesis

The hypothesis is that achieving the endpoints is more likely with at least one of these interventions (CVD PRS alone or in combination with digital technology) compared to individuals who only undergo the traditional CVD risk assessment at the beginning of the study, as well as compared to individuals who receive traditional risk assessment along with the use of smart band.

## Methods and design

This study protocol was written according to SPIRIT (Standard Protocol Items: Recommendations for Interventional Trials), a guideline that defines standard protocol items for clinical trials and is widely endorsed as an international standard for trial protocols (10, 11).

## Study design

**Figure 1:**
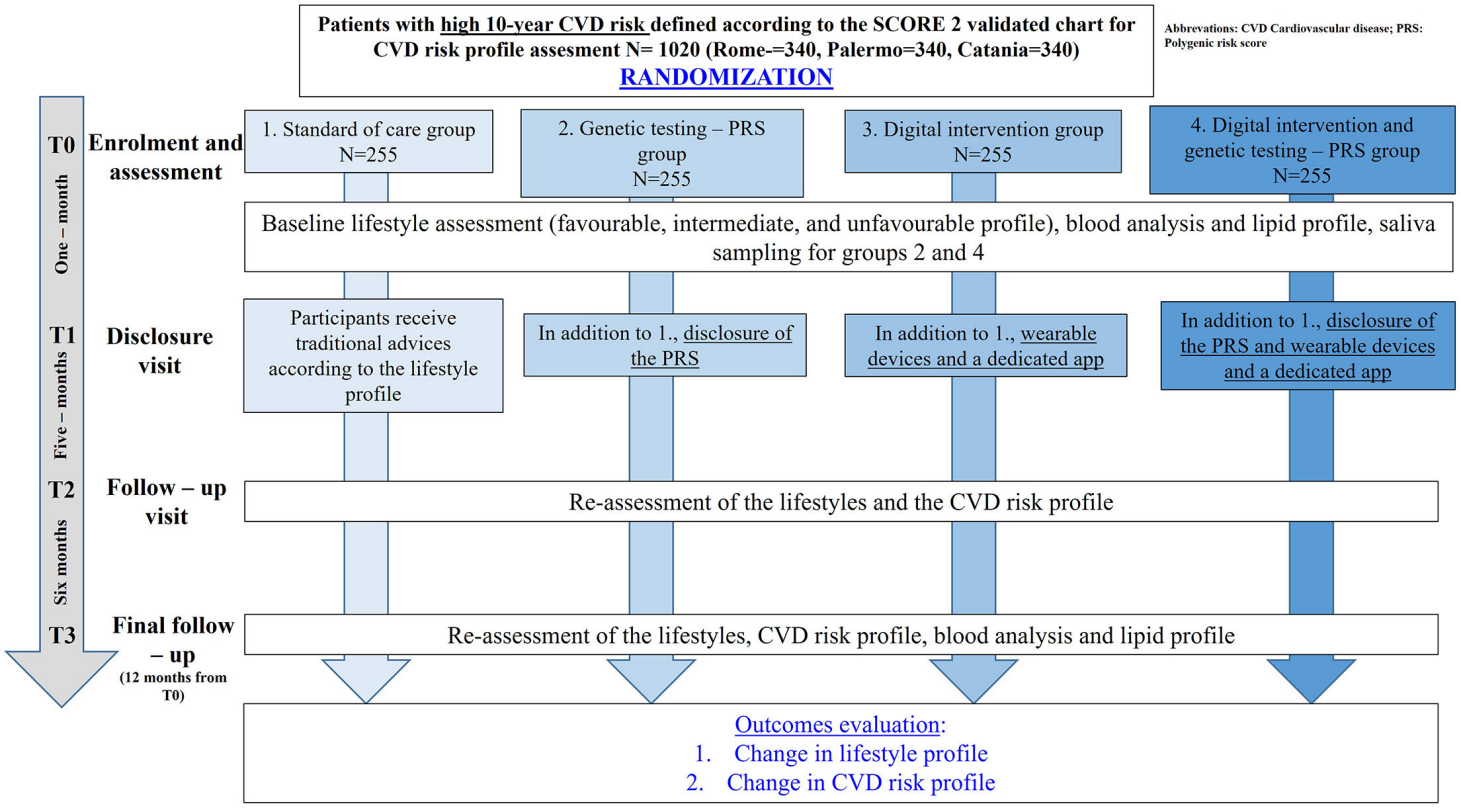
The INNOPREV trial.

This is an RCT with four parallel arms, as illustrated in Figure 1. Participants will be randomly assigned to the following intervention groups:

A. No Intervention—Traditional CVD risk assessment.
B. Traditional CVD risk assessment plus Genetic testing (CVD PRS).
C. Traditional CVD risk assessment plus Digital intervention (use of an app and a smart band).
D. Traditional CVD risk assessment plus Digital intervention (use of an app and a smart band) plus genetic testing (CVD PRS).

A detailed explanation of each of the interventions can be found in the “Interventions” section.

## Participants, study setting and recruitment

### Participants, inclusion and exclusion criteria

The study will include individuals between the ages of 40 and 69 years who do not have established CVDs, diabetes mellitus, or familial hypercholesterolemia. These individuals should have a high 10-year CVD risk ranging from 2.5 to 10% based on the SCORE 2 charts (12).

### Study setting

The trial will take place in three regions of Italy: Catania and Palermo (Southern Italy) and Rome (Central Italy). The enrollment of individuals at high cardiovascular risk will occur in different settings per site: primary care ambulatories (Rome), general practitioners' offices (Catania), and a seasonal vaccination center (Palermo).

### Recruitment

The recruitment process will span a period of 8 months, and participants will be followed for a total of 12 months. During the initial medical visit, the physician will inquire about the person's interest in participating in the study. If the person agrees, they will be enrolled, and the planned interventions will be implemented. To ensure effective recruitment, the study will employ a comprehensive advertising campaign targeting the population. This campaign will utilize traditional marketing strategies, including billboards, flyers, and brochures, while also leveraging digital platforms with targeted ads on social networks. These efforts aim to reach and engage a diverse range of potential participants.

## Randomization and masking

Participants will be allocated to one of the four groups (1:1:1:1) according to a restricted randomization procedure (13). A study investigator (TP) will generate the allocation sequences using both stratification and permuted blocks with random block sizes and block order. Stratification factors will include the three settings per site: primary care ambulatory, general practitioners' office, and seasonal vaccination center. The table of allocation will not be disclosed to ensure concealment and the randomization will be provided through Research Electronic Data Capture (RedCap) web application. The assignment to intervention will be unmasked to all trial participants: patients, research staff, and the medical team will be only aware of study group assignment after randomization procedures.

## Interventions

All participants will undergo a standardized in-person consultation to receive medical advice for CVD prevention. The advice will be provided in written form and will follow the European Guidelines on cardiovascular risk prevention (14).

### Digital intervention

Participants in groups C and D will be provided with smart bands equipped with sensors to monitor their daily physical activity levels, calories burned, sleep patterns, and heart rate. The collected data will be accessible to the participants through a related mobile app (15) where they can view trends in heart rate, the number of steps taken, daily calorie burn, and the quality of night-time sleep. The app will provide personalized feedback and insights based on the recorded information, such as reminders to achieve a certain number of steps each day to maintain an active lifestyle, in order to reach the optimal suggested number of 6,000–8,000 steps per day (16).

### Genetic testing

Participants assigned to the groups B and D will undergo genetic testing to evaluate their CVD genetic risk profile using a commercially available genome-wide PRS. At the time of enrollment, participants will receive a salivary swab to obtain genomic DNA for analysis. The PRS analysis will be conducted using the Illumina microarray technology, which is a cost-effective genotyping approach for disease and population genetics studies (17). Microarray analysis will generate data on millions of genetic variants per individual, and any gaps between these variants will be filled using imputation analysis. The imputation process compares the sequencing results with a reference dataset, leveraging reference genomes from the 1,000 Genomes Project (18). A modified version of the GLIMPSE algorithm will be utilized for imputing genomic data (19). Based on the combined results, individuals will be classified into three main genetic risk categories for CVD: High risk, Intermediate risk, and Normal risk.

## Study procedure

Eligible individuals accessing primary care ambulatories, general practitioners' offices, and vaccination centers will be invited to participate in the study. They will receive detailed information both orally and through an informative sheet.

Upon enrollment (T0), participants will sign the informed consent form administered by the physicians and will be randomized to one of the four intervention groups through the REDCap (Research Electronic Data Capture) system (19), which will also record all participants information during the study. Participants, research staff, and physicians will be aware of the study group assignment after randomization.

At T0, all participants will undergo a comprehensive assessment and cardiovascular evaluation. This will involve completing questionnaires covering various aspects such as socioeconomic status, area of residence, and key lifestyle factors including smoking, alcohol consumption, dietary patterns, sleep patterns, and physical activity. The lifestyle assessment utilizes the Life's Essential 8 questionnaire, categorizing each participant into a favorable, intermediate, or unfavorable individual lifestyle pattern (20). This metric was recently introduced by the American Heart Association (AHA) for evaluating cardiovascular wellbeing throughout elements such as physical activity, body mass index, and blood pressure, diet, nicotine exposure, blood lipids and blood glucose, and sleep habits (20). The domain of diet has been explored by a new method for the evaluation of individual diet quality as a modified Mediterranean Eating Pattern for Americans (MEPA). This approach emphasizes individuals' dietary patterns and whole food consumption over specific nutrients, promoting its application in clinical and research contexts.

Additionally, participants will be asked questions focusing on psychological status, as well as their knowledge and attitudes toward CVDs, their knowledge and awareness of personalized medicine, genetic testing and PRS, and their acceptance of wearable devices (21–23). All questionnaires are provided in Appendix 1.

The cardiovascular evaluation will include measurements of blood pressure, heart rate, objective examination and cardiovascular risk assessment using SCORE 2 charts, incorporating essential variables such as age, gender, systolic blood pressure, total cholesterol, HDL cholesterol, and smoking status (12). Blood analysis will also be conducted, assessing parameters such as total cholesterol, HDL and LDL cholesterol, triglycerides, high sensitivity C-Reactive Protein (CRP), glycated hemoglobin, and/or glycemia.

Participants in Groups B and D will also undergo a salivary swab for PRS analysis.

Subsequently, participants will have a disclosure visit (T1) after 1 month, a follow-up visit (T2) after 5 months, and a final follow-up visit (T3) at 12 months.

During the disclosure visit (T1), participants in all groups will receive an explanation of their lifestyle pattern and cardiovascular risk profile based on SCORE 2 charts, along with written preventive advice for risk reduction and lifestyle changes. Participants in the digital intervention groups (C, D) will also receive an app and a smart band to monitor various health data. A comprehensive demonstration during smart band delivery, along with written instructions on its usage, will be provided to ensure participants consistently utilize the smart band throughout the study, with the recommendation for daily application (24–26). Participants in the PRS groups (B, D) will receive the disclosure of their genetic CVD risk profile.

At both follow-up visits (T2, T3), the comprehensive questionnaire will be administered again to assess changes in lifestyles and psychological status. Any challenges with smart band usage will also be identified. At the final visit (T3), the cardiovascular risk profile will be recalculated, and participants will undergo a final full assessment, evaluation of the lifestyle category, and blood test to evaluate changes in cholesterol levels and other parameters.

## Outcome measures

### Primary outcomes

#### Lifestyle change

The Life's Essential eight questionnaire (20) will be administered at T0, T2, and T3 to assess changes in lifestyle category (favorable, intermediate, or unfavorable) compared to baseline.

### Secondary outcomes

#### Cardiovascular risk change

The SCORE 2 charts will be used to calculate cardiovascular risk at T0, T2, and T3, allowing for the evaluation of changes in risk compared to baseline.

#### Lipid profile

Blood analysis will be conducted at T0 and T3 to measure changes in lipid levels compared to baseline. Parameters examined will include total cholesterol, HDL and LDL cholesterol, and triglycerides.

By assessing these measures at different time points throughout the trial, the study will evaluate the impact and progress achieved by participants over the course of the intervention.

### Data collection and management

The study data will be collected and managed using the REDCap electronic data capture tools (27). Access to the REDCap web platform and data entry/management will be restricted to individuals officially registered as study investigators or data managers. They will receive user login credentials to ensure secure access to the system. Each study unit will enter the data into the eCRFs in a pseudonymized form, following established protocols and within the designated timeframes after each participant visit.

### Statistical methods

#### Sample size estimation

Based on a conservative assumption, considering that this is the first study in this context, we anticipate that 15% of participants in group D will transition from unfavorable to intermediate lifestyle patterns during the follow-up period, compared to 10% in group C, 8% in group B, and 5% in group A. To achieve a statistical power of at least 80% with a significance level of 0.05, while accounting for a potential withdrawal rate of 30%, a total of 1,020 participants will need to be recruited, with 255 participants in each group. This sample size allows us to have a statistical power >80% to detect a difference of 5 mg/dL in LDL cholesterol (our secondary outcome) with a standard deviation of 15 mg/dL and a significance level of 0.05%, as tested in previous studies (28). An interim analysis will be conducted when 50% of the participants will be recruited to assess whether the sample size remains adequate for detecting the anticipated differences in lifestyle patterns and LDL cholesterol levels.

#### Statistical methods for primary and secondary outcomes

The primary analysis will be based on the intention-to-treat principle, where all randomized participants will be analyzed according to their allocated study arm. Baseline characteristics of the four arms will be summarized using descriptive statistics. The baseline characteristics of those who withdraw will be assessed against those who remain in the study.

Groups D, C, and B will be compared with group A to evaluate the marginal effect of adding at least one intervention or both. The analysis will employ adjusted mixed-effect models for repeated measures to assess significant differences in lifestyle patterns, lipid, and CVD risk profiles from baseline to the various follow-up time points (T).

Furthermore, an analysis of the primary outcome will be conducted in a per-protocol fashion to account for non-compliance with uptake of the CVD PRS or use of the app/smart band evaluated at T2 and T3, as well as missed appointments for the complete CVD risk result, as prescribed by their allocated intervention.

#### Subgroup analyses

We will conduct a comprehensive subgroup analysis to explore potential moderating and mediating effects based on participant characteristics. The subgroup analysis will encompass sociodemographic characteristics (age above or below 55 years, gender, and marital status), ethnicity, socioeconomic status (occupation and related variables), education level, PRS levels (high, intermediate, normal), lifestyle category at baseline (favorable, intermediate, or unfavorable), attitudes toward the use of digital technologies (present/absent), knowledge toward CVDs (present/absent), and psychological status at baseline. The primary objective of this subgroup analysis is to investigate whether the efficacy of the interventions varies across these identified participant subgroups. We aim to understand how sociodemographic factors, genetics, and baseline lifestyle categories may moderate or mediate the effects of the interventions on behavior change and modification of CVD risk profiles.

Statistical analyses will be conducted using STATA (StataCorp, USA) and R (https://www.r-project.org/).

## Discussion

This study investigates the efficacy of providing CVD PRS information, alone or in combination with an app and a smart band, on changes in lifestyle patterns and modification of CVD risk profiles.

The aim is to determine whether citizens are more inclined to modify their lifestyle if they are aware of their cardiovascular genetic risk profile and are monitored by an app and smart band that display their levels of physical activity and related parameters.

To date, limited research has explored this aspect, making the INNOPREV trial one of the largest studies in this field.

Previous studies have shown that the provision of personalized genetic information can positively influence screening behaviors and medication adherence for individuals at risk of familial cancers, particularly those with high-penetrance genetic variants (29–31). However, the impact of genetic risk communication on the adoption of complex lifestyle behaviors, such as dietary modification, has not been well-supported by existing evidence (32–37). Systematic reviews investigating the effect of communicating genetic risk on lifestyle modification for cardiometabolic disorders have found no evidence of improved dietary or exercise behaviors (33, 35). Although the studies included in these reviews differ in design characteristics and interventions used, current empirical data consistently suggest that receiving genetic feedback has no significant impact on outcomes, regardless of whether genetic feedback was provided for one gene, multiple genes, or in the form of PRS. Some evidence, however, exists regarding higher initiation of treatments for cholesterol control, such as statins, in groups with genetic information compared to control groups (34, 37).

The limitations of previous studies may contribute to the failure of detecting effects associated with personalized genetic feedback. These limitations include being underpowered in detecting small effect sizes, biased samples or lack of generalizability (33), uninformative control groups, short duration of follow-up (36), poorly designed interventions and the use of self-reporting measures as outcomes (33, 35). Additionally, none of the clinical studies examined potential mediators of behavior change relevant to genetic testing, such as genetic and health literacy and baseline motivations (38). Nevertheless, positive findings from previous studies demonstrate that genetic information for complex polygenic traits like CVD can be effectively provided in busy clinical practices without significant adverse psychological effects (32, 36).

The INNOPREV trial will be the largest trial to date with objective outcome measures, specifically measuring changes in lipid levels. Participants will be also characterized to capture risk comprehension and initial levels of motivation, enabling health professionals to better tailor risk feedback.

The larger sample size of our study enhances statistical power and generalizability, making it a significant contribution to the field. Additionally, the inclusion of digital tools, such as apps, to monitor lifestyle changes provides an advantage in facilitating continuous monitoring, personalized feedback, and motivation for adherence to risk-reducing strategies.

However, some limitations of the use of the modified MEPA as a proxy for dietary quality need to be discussed. First, MEPA is a rapid tool developed to be applicable in clinical practice. Its value resides in its feasibility and focus on individuals' eating patterns and whole foods rather than nutrients. Compared to more structured dietary assessment, MEPA is less comprehensive than the tools generally used in epidemiological research. Second, the questionnaire is self-administered, and therefore, inaccurate responses due to misinterpretation of the questions cannot be excluded. Additionally, the tool has not been used and validated in the Italian population. Cultural differences, translation accuracy, and semantic equivalence can influence the accuracy and relevance of the instrument. Despite these limitations in the dietary assessment, the use of Life Essential's 8 and its domains is important to assess lifestyle-related changes in the study population. Its increasing use for cardiovascular health measurement ensures wider comparability of our results. Moreover, PRS may suffer from poor generalizability to different populations, which could potentially impact the generalizability of the study conclusions. However, the PRS analysis will be conducted using a cutting-edge commercially available genome-wide PRS for CVD genetic risk profiling. Finally, our trial is conducted within a specific cultural and healthcare context in Italy, and therefore, our conclusions may be limited to populations with similar cultural backgrounds. Primary prevention is a crucial goal for healthcare providers, as it can result in remarkable reductions in event rates when applied optimally. However, achieving behavior change and medication adherence in clinical practice can be challenging. Behavior change is a complex process (38). Simply providing PRS information without considering the subjective perception of CVDs, as well as the perceived advantages and barriers toward preventive actions, may diminish the potential impact of receiving genetic risk information on behavior change.

The INNOPREV trial aims to comprehensively evaluate these aspects, including the implementation of a patient-centered approach, which is crucial for ensuring the adoption of preventive lifestyle choices for CVD in the medium and long term (35). By delivering personalized information to patients about their individual risk profile and engaging them in meaningful discussions about the available strategies to reduce their CVD risk, we seek to empower individuals to make informed decisions that align with their preferences and goals. This approach can increase the likelihood of sustained behavior change and adherence to risk-reducing strategies over time, ultimately leading to better cardiovascular health outcomes.

## Ethics statement

This trial has been approved by the Ethics Committee of the Fondazione Policlinico Universitario Agostino Gemelli (comitato.etico@policlinicogemelli.it), with the approval number: 5506. Furthermore, the trial has been approved by the Local Ethics Committee Catania 2 with the protocol number: 149 C.E. (101/CECT2) and by the Local Ethics Committee Palermo 1 with the approval number CE 150109. This trial has been registered on Clinical Trial Gov., with the identifier: NCT05883878.

## Author contributions

RP: Writing – original draft, Conceptualization, Formal analysis, Funding acquisition, Investigation, Methodology, Project administration, Supervision, Writing – review & editing. AP: Writing – original draft, Formal analysis, Investigation, Methodology, Writing – review & editing. AA: Formal analysis, Funding acquisition, Investigation, Methodology, Project administration, Supervision, Writing – review & editing. CdW: Formal analysis, Funding acquisition, Investigation, Methodology, Project administration, Supervision, Writing – review & editing. WM: Formal analysis, Funding acquisition, Investigation, Methodology, Project administration, Supervision, Writing – review & editing. LR: Writing – original draft, Formal analysis, Investigation, Methodology, Writing – review & editing. MBi: Formal analysis, Investigation, Methodology, Writing – review & editing. AM: Formal analysis, Investigation, Methodology, Writing – review & editing. SFa: Formal analysis, Investigation, Methodology, Writing – review & editing. MP: Formal analysis, Investigation, Methodology, Writing – review & editing. DT: Formal analysis, Investigation, Methodology, Writing – review & editing. MDP: Formal analysis, Investigation, Methodology, Writing – review & editing. RA: Formal analysis, Investigation, Methodology, Writing – review & editing. MW: Formal analysis, Investigation, Methodology, Writing – review & editing. TP: Formal analysis, Investigation, Methodology, Supervision, Writing – review & editing. MBa: Formal analysis, Investigation, Methodology, Writing – review & editing. RM: Formal analysis, Investigation, Methodology, Writing – review & editing. GF: Formal analysis, Investigation, Methodology, Writing – review & editing. AT: Formal analysis, Investigation, Methodology, Writing – review & editing. FT: Formal analysis, Investigation, Methodology, Writing – review & editing. GM: Formal analysis, Investigation, Methodology, Writing – review & editing. DD: Formal analysis, Investigation, Methodology, Writing – review & editing. SFr: Formal analysis, Investigation, Methodology, Writing – review & editing. AS: Formal analysis, Investigation, Methodology, Writing – review & editing. GL: Formal analysis, Funding acquisition, Investigation, Methodology, Project administration, Supervision, Writing – review & editing. SB: Writing – original draft, Writing – review & editing, Conceptualization, Formal analysis, Funding acquisition, Investigation, Methodology, Project administration, Supervision.
