# Correction: Efficacy of polygenic risk scores and digital technologies for INNOvative personalized cardiovascular disease PREVention in high-risk adults: protocol of a randomized controlled trial

**DOI:** 10.3389/fpubh.2025.1696816

**Published:** 2025-09-17

**Authors:** Roberta Pastorino, Angelo Maria Pezzullo, Antonella Agodi, Chiara de Waure, Walter Mazzucco, Luigi Russo, Martina Bianchi, Alessandra Maio, Sara Farina, Martina Porcelli, Diego Maria Tona, Matteo Di Pumpo, Rosarita Amore, Malgorzata Wachocka, Tina Pasciuto, Martina Barchitta, Roberta Magnano San Lio, Giuliana Favara, Antonino Tuttolomondo, Fabio Tramuto, Gaia Morello, Daniele Domenico De Bella, Santo Fruscione, Anna Severino, Giovanna Liuzzo, Stefania Boccia

**Affiliations:** ^1^Section of Hygiene, Department of Life Sciences and Public Health, Università Cattolica del Sacro Cuore, Rome, Italy; ^2^Department of Woman and Child Health and Public Health, Fondazione Policlinico Universitario A. Gemelli IRCCS, Rome, Italy; ^3^Department of Medical and Surgical Sciences and Advanced Technologies “GF Ingrassia”, University of Catania, Catania, Italy; ^4^Azienda Ospedaliero Universitaria Policlinico “G. Rodolico-San Marco”, Programme: Hospital Epidemiology and Translational Research: Epidemiological Surveillance, Risk Assessment and Control, Catania, Italy; ^5^Department of Medicine and Surgery, University of Perugia, Perugia, Italy; ^6^Azienda Ospedaliera Universitaria Policlinico “Paolo Giaccone” di Palermo, Palermo, Italy; ^7^Dipartimento PROMISE, Università degli Studi di Palermo, Palermo, Italy; ^8^Research Core Facilty Data Collection G-STeP, Fondazione Policlinico Universitario A. Gemelli IRCCS, Rome, Italy; ^9^Department of Cardiovascular Sciences, Fondazione Policlinico Universitario A. Gemelli—IRCCS, Rome, Italy; ^10^Department of Cardiovascular and Pulmonary Sciences, Università Cattolica del Sacro Cuore, Rome, Italy

**Keywords:** cardiovascular disease, polygenic risk score (PRS), smart bands, personalized prevention, trial

In the published article, there was an error in the Funding statement. The Funding statement incorrectly attributed funding to the Italian Ministry of Health instead of the correct source. The correct Funding statement appears below.

