# Supplementary material for: Efficacy of polygenic risk scores and digital technologies for INNOvative personalized cardiovascular disease PREVention in high-risk adults: protocol of a randomized controlled trial
Source: Front Public Health. 2024 Jun 14;12:1335894. doi: 10.3389/fpubh.2024.1335894 (PMC11211566; doi:10.3389/fpubh.2024.1335894)
Supplement: Supplementary file 1 [file Data_Sheet_1.PDF]

Appendix

INNOPREV questionnaire

1. SECTION 1 2
1.1. PERSONAL DATA 2
1.2 What is your or degree of education? 2
1.3 What is your marital status? 2
1.4 Where do you live? 2
1.5 What is your employment status? 2
1.6 Do you have a smartphone 3
1.2 DIET 3
1.3 PHYSICAL ACTIVITY 4
1.4 NICOTINE EXPOSURE 5
1.5 SLEEPING TIME 5
2. SECTION 2 5
2.1 PERSONAL HISTORY 5
2.2 FAMILY BACKGROUND 6
2.3 PERCEIVED STRESS 6
2.4 REACTION TO GENETIC TESTING 7
3. SECTION 3 8
3.1 BLOOD PRESSURE (in mmHg) 8
3.2 CHOLESTEROL (in mg/dL) 8
3.3 GLICEMIA (mg/dL) or HbA1c (%) 8
3.4 BODY MASS INDEX 8

1. SECTION 1

1.1. PERSONAL DATA

1.1 Biographical Data
Gender: M / F
Age: …… years old
Nationality: Italian or Other (if other, please specify) ……
Town/City: ……
Home Address: ……
Enter your postal code: ……

1.2 What is your or degree of education?

No degree
Elementary schooling
Middle school diploma
High school diploma
Bachelor's degree
Master's degree
PhD degree
Don't know/Prefer not to answer

1.3 What is your marital status?

Single
Boyfriend/girlfriend
Civil partner
Married
Separated/divorced
Widow/widower
Don't know/Prefer not to answer

1.4 Where do you live?

In a large city (population more than 200,000)
In a city (less than 200,000 inhabitants)
In a rural area (e.g., countryside, mountains)
Has your residence changed often in recent years?
Don't know/Prefer not to answer

1.5 What is your employment status?

Don't know/Prefer not to answer
Full time employment
Working less than five days a week
Part time employment
Retired due to disability
Retired due to age limit
Partially retired
Unemployed
Stay at home dad/mom
Other (describe below)

1.6 Do you have a smartphone

Yes / No

1.2 DIET

1. How many tablespoons of olive oil do you consume (including for both dressing and cooking)?
Any number greater than or equal to 0 is allowed.

2. How many servings (a serving is approximately 200 grams) of vegetables do you consume **per day**?
Any number greater than or equal to 0 is allowed.

3. How many servings of fruit (a serving is approximately 150 grams, e.g.: one apple) do you consume **per day**?
Any number greater than or equal to 0 is allowed.

4. How many servings of bread, pasta, rice or cereals do you consume **per day**? (For a serving of bread, it is 50 grams, for pasta or cereals, it's 80 grams)
Any number greater than or equal to 0 is allowed.

5. How many times **per day** do you consume sweets (e.g., cookies, pastries, slices of cake, croissants and brioches)?
Any number greater than or equal to 0 is allowed.

6 A: How many alcoholic units do you consume **per day**? (FOR WOMEN)
An alcoholic unit corresponds to 12 grams of pure alcohol and is equivalent to:
a glass of wine (125 ml at 12°)
a can of beer (330 ml at 4.5°)
an aperitif drink (80 ml at 38°)
a shot of spirits (40 ml at 40°)
Any number greater than or equal to 0 is allowed.

6 B: How many alcoholic units do you consume **per day**? (FOR MEN)
(One alcoholic unit corresponds to 12 grams of pure alcohol and is equivalent to:
a glass of wine (125 ml at 12°)
a can of beer (330 ml at 4.5°)
an aperitif drink (80 ml at 38°)
a shot of spirits (40 ml at 40°)
Any number greater than or equal to 0 is allowed.

7. How many servings (a serving is approximately 80 grams) of broad leafy vegetables (such as spinach) do you consume **per week**?
Any number greater than or equal to 0 is allowed.

8. How many servings (a serving is approximately 80 grams) of berries do you consume **per week**?
Any number greater than or equal to 0 is allowed.

9. How many servings (a serving is approximately 100 grams) of red meat do you consume **per week**?
Any number greater than or equal to 0 is allowed.

10. How many servings (a serving is approximately 150 grams) of fish (not fried) do you consume in **per week?**
Any number greater than or equal to 0 is allowed.

11. How many servings (a serving is approximately 100 grams) of chicken (not fried) do you consume **per week?**
Any number greater than or equal to 0 is allowed.

12. How many servings of cheese (a serving is at least 50 grams) do you consume **per week?**
Any number greater than or equal to 0 is allowed.

13. How many servings (a serving is approximately 10 grams) of butter do you consume **per week**?
Any number greater than or equal to 0 is allowed.

14. How many servings of fresh or canned legumes (approximately 150 grams) or dried legumes (approximately 50 grams) legumes do you consume **per week**?
Any number greater than or equal to 0 is allowed.

15. How many servings (a serving being approximately 30 grams) of dried fruits (e.g., almonds, hazelnuts, walnuts) do you consume **per week**?
Any number greater than or equal to 0 is allowed.

16. How many meals do you consume in fast food restaurants (example: McDonald's or Burger King) **per week**?
Any number greater than or equal to 0 is allowed.

1.3 PHYSICAL ACTIVITY

1a. How many minutes of intense intensity physical activity (example: e.g., lifting weights, heavy yard work, aerobic activities such as running or bicycling at high speed) do you perform per week?
Any number greater than or equal to 0 is allowed. (in minutes)

1b. How many minutes of moderate-intensity physical activity (example: e.g., carrying light weights, bicycle rides at a regular speed, gym activities, garden work, prolonged physical work at home, walking at a brisk pace) do you do per week?
Any number greater than or equal to 0 is allowed. (in minutes)

NB: for the final score the two questions will be merged.

1.4 NICOTINE EXPOSURE

1.Are you a smoker?
Never been a smoker
I am currently a smoker (if yes, please answer in the box below):
Ø how many cigarettes per day? (a number greater than 0)
Ø for how many years? (a number greater than 0)
Former smoker: (if yes, open the boxes below)
Ø Less than one year ago or currently using (open the box below):
§ Heated tobacco cigarettes (Iqos, Glo etc.).
§ electronic cigarettes
§ Other (please specify)
Ø From 1 to 5 years
Ø More than five years ago

1.5 SLEEPING TIME

1. On average, how many hours do you sleep in a 24-hour period?
Any number greater than or equal to 0 (in hours) is allowed.

2. SECTION 2

2.1 PERSONAL HISTORY

1. How many liters of water do you drink in a day?
Less than half a liter / A liter / One and a half liters / Two liters / Two and a half liters

2. How often do you add salt to your food at the table?
Never or rarely / Rather often /Always or very often

3. Do you find yourself very thirsty, particularly after a meal?
Never or rarely / Rather often /Always or very often

4. Do you consume medication on a daily basis?
Yes / No

If yes please indicate which ones (open answer)
Anxiolytics
Antihypertensives
Anti-inflammatories
Antihypertensives
Cholesterol-lowering drug
Other (indicate which)

FOR WOMEN ONLY
Menopause:
Yes / No / Don't know

2.2 FAMILY BACKGROUND

Among your family members (parents, siblings, children) has anyone suffered from one or more of the following illnesses?

5. Early cardiovascular disease (heart attack, angina, cardiac ischemia, sudden cardiac death, angioplasty (balloon/stent) or coronary artery bypass), respond:
No / Yes / Don't know

6. Early cerebrovascular disease (stroke, hemorrhagic stroke, cerebral ischemia, transient cerebral ischemia (also called TIA), respond:
No / Yes / Don't know

Among your family members, has someone been affected by:
7. High cholesterol (in parents, brothers, sisters, children)
No / Yes / Don't know

8. Diabetes (in at least one of the birth parents)
No / Yes / Don't know

9. Diabetes (in brothers, sisters, children)
No / Yes / Don't know

10. Diabetes (in grandparents, aunts and uncles, cousins)
No / Yes / Don't know

11. Hypertension (father)
No / Yes / Don't know

12. Hypertension (mother)
No / Yes / Don't know

2.3 PERCEIVED STRESS

13. In the past month, how often have you felt unable to think clearly and control yourself due to strong emotions because something unexpected happened?
Never / Almost never /Sometimes / Quite often / Very often

14. In the past month, how often have you felt unable to have control over important aspects of your life?
Never / Almost never /Sometimes / Quite often / Very often

15. In the past month, how often have you felt nervous or "stressed"?
Never / Almost never /Sometimes / Quite often / Very often

16. In the past month, how often have you felt confident about your ability to handle your personal problems?
Never / Almost never /Sometimes / Quite often / Very often

17. In the past month, how often did you feel that things were going according to your plans?
Never / Almost never /Sometimes / Quite often / Very often

18. In the past month, how often have you felt that you could not keep up with all the things you had to do?
Never / Almost never /Sometimes / Quite often / Very often

19. In the past month, how often have you felt able to control what is irritating you in your life?
Never / Almost never /Sometimes / Quite often / Very often

20. In the past month, how often have you felt in control of the situation?
Never / Almost never /Sometimes / Quite often / Very often

2.4 REACTION TO GENETIC TESTING

If you received a genetic test result that indicated a higher risk of cardiovascular disease……

21. I would feel that there is nothing I can do to prevent cardiovascular diseases.
Not at all / Somewhat / Moderately / Very / Very much

22. I would be worried about having cardiovascular disease
Not at all / Somewhat / Moderately / Very / Very much

23. I would regret taking the test
Not at all / Somewhat / Moderately / Very / Very much

24. I would feel depressed about the result
Not at all / Somewhat / Moderately / Very / Very much

25. I would be happy to know the result
Not at all / Somewhat / Moderately / Very / Very much

26. Have you talked to your family or friends about their risk of cardiovascular disease?
No, I haven’t talked to anyone.
Yes, I’ve had conversations with them but have not encouraged them to consult a doctor.
Yes, I’ve had conversations with them and have encouraged them to see a doctor.
Yes, after talking to me someone I know saw a doctor.

3. SECTION 3

3.1 BLOOD PRESSURE (in mmHg)

Systolic blood pressure: ……
Diastolic blood pressure: ……
Do you take blood pressure lowering medications? Please put yes or no: ……
(If yes, specify which ones, open-ended question) ……

3.2 CHOLESTEROL (in mg/dL)

Non-HDL cholesterol (non-high-density cholesterol, which comprises the sum of total cholesterol minus HDL cholesterol): ……

Do you take cholesterol-lowering medications? Please answer yes or no: ……
If yes, specify which ones (open-ended question) ……

LAST EXAM DATE (if under six months) (open-ended section)

3.3 GLICEMIA (mg/dL) or HbA1c (%)

Do you have diabetes?: ……
Fasting blood glucose: ……
Glycated hemoglobin HbA1c: ……

LAST EXAM DATE (if under six months) (open-ended section)

3.4 BODY MASS INDEX

Height (in cm): ……
Weight (in kg): ……
Based on these values the BMI will be automatically calculated: ……

VALUES AND PREFERENCES QUESTIONNAIRE

HBCVD

(Tovar EG, Rayens MK, Clark M, Nguyen H. Development and psychometric testing of the Health Beliefs Related to Cardiovascular Disease Scale: preliminary findings. J Adv Nurs. 2010 Dec;66(12):2772-84. doi: 10.1111/j.1365-2648.2010.05443.x. Epub 2010 Sep 10. PMID: 20831570.)

The response format for each item is a 4-point Likert scale
1 = Strongly Disagree; 2 = Disagree; 3 = Agree; 4 = Strongly Agree

1. It is likely that I will suffer from a heart attack or stroke in the future
2. My chances of suffering from a heart attack/stroke in the next few years are great
3. I feel I will have a heart attack or stroke sometime during my life
4. Having a heart attack or stroke is currently a possibility for me
5. I am concerned about the likelihood of having a heart attack/stroke in the near future
6. Having a heart attack or stroke is always fatal
7. Having a heart attack or stroke will threaten my relationship with my significant other
8. My whole life would change if I had a heart attack or stroke
9. Having a heart attack or stroke would have a very bad effect on my sex life
10. If I have a heart attack or stroke, I will die within 10 years
11. Increasing my exercise will decrease my chances of having a heart attack or stroke
12. Eating a healthy diet will decrease my chance of having a heart attack or stroke
13. Eating a healthy diet and exercising for 30 minutes most days ... to prevent a heart attack/stroke
14. When I exercise I am doing something good for myself
15. When I eat healthy I am doing something good for myself
16. Eating a healthy diet will decrease my chances of dying from cardiovascular disease
17. I don’t know appropriate exercises to perform to reduce my risk of developing cardiovascular disease
18. It is painful for me to walk for more than 5 minutes
19. I have access to exercise facilities and/or equipment
20. I have someone who will exercise with me
21. I don’t have time to exercise for 30 minutes a day on most days of the week
22. I don’t know what is considered a healthy diet that would prevent me from developing cardiovascular disease
23. I don’t have time to cook meals for myself
24. I cannot afford to buy healthy food
25. I have more important problems than worrying about diet and exercise

The Vanderbilt PRS-KS

(Stubbs D, Hooker G, Li Y, Richter L, Bick A, Development and Validation of the Vanderbilt PRS-KS, an Instrument to Quantify Polygenic Risk Score Knowledge, Genetics in Medicine Open (2023), doi: https://doi.org/10.1016/j.gimo.2023.100822.)

1. Polygenic risk scores are based on genetic changes in more than one gene
2. All people who receive a high-risk result on a polygenic risk score for a disease will develop that disease.
3. A polygenic risk score can combine other health determinants beyond genetics.
4. Polygenic risk scores have the same accuracy regardless of the disease being testing for.
5. Polygenic risk scores have the same accuracy for all people, regardless of their racial or ethnic background.
6. If you receive a high polygenic risk score result, your children will have a high polygenic risk result.
7. Your polygenic risk score could find a decreased risk for disease.

UTAUT

(Uncovska M, Freitag B, Meister S, Fehring L. Patient Acceptance of Prescribed and Fully Reimbursed mHealth Apps in Germany: An UTAUT2-based Online Survey Study. J Med Syst. 2023 Jan 27;47(1):14. doi: 10.1007/s10916-023-01910-x. PMID: 36705853; PMCID: PMC9880914)

The response format for each item is a 4-point Likert scale
1 = Strongly Disagree; 2 = Disagree; 3 = Agree; 4 = Strongly Agree

The questionnaire addressing the use of digital technologies has two different parts, one on common knowledge and one (UTAUT) specific to SMARTBAND knowledge to be administered at T0 to all participants. At T2 and T3, the same questionnaire will be administered again only to arms 2 and 4.

Common knowledge

Q: How do you rate your IT skills? (Answered as single-choice question)
A: a. Very high b. High c. Average d. Low e. Very low

Q: Do you use the following devices and, if yes, what is the main purpose?
A:
Computer: Yes/No; Work/Free Time
Tablet: Yes/No; Work/Free Time
Smartphone: Yes/No; Work/Free Time

Q: Have you ever used digital applications (apps) for health?
A: Yes; No

UTAUT

Performance expectations

I expect/feel that the use of the wristband or SMARTBAND is useful in my daily life
I expect/feel that the use of the wristband or SMARTBAND increases my chances of achieving health targets/controlling my lifestyles
I expect/feel that the use of the wristband or SMARTBAND help in monitoring my lifestyle
I expect/feel that the use of the wristband or SMARTBAND increases my ability to manage my lifestyle
I expect/feel that the use of the wristband or SMARTBAND increases the quality of care

Effort expectations

Learning to use the digital technology/the wristband or SMARTBAND is/was easy for me

Interaction with the digital technology/the bracelet or SMARTBAND is clear and understandable

Using the digital technology/the bracelet or SMARTBAND requires/has required little training

Using the digital technology/wristband or SMARTBAND is easy

I find it easy/it was easy for me to become proficient in using the digital technology/wristband or SMARTBAND

Social influence

People who are important to me think I should use digital technology/the bracelet or SMARTBAND
People who influence my behavior think I should use the digital technology/the bracelet or SMARTBAND
Digital technology/the bracelet or SMARTBAND will be used by many people in the future

Facilitating conditions

I have the necessary knowledge to use the digital technology/wristband or SMARTBAND
My use of the digital technology/the bracelet or SMARTBAND is compatible with other technologies I use
I can get help from others when I have difficulty using the digital technology/the bracelet or SMARTBAND
